# Editorial: Re-visiting risk factors for cardiometabolic diseases: towards a new epidemiological frontier

**DOI:** 10.3389/fpubh.2026.1821553

**Published:** 2026-06-05

**Authors:** Yashendra Sethi, Inderbir Padda, Mayur S. Parmar, Linwei Tian, Gurpreet Johal

**Affiliations:** 1 PearResearch, Dehradun, India; 2 National Institute of Cardiometabolic Excellence (NICE), Vigyaved Healthcare, Dehradun, India; 3 Subharti Medical College, Meerut, India; 4 Nova Southeastern University Dr. Kiran C. Patel College of Osteopathic Medicine, Clearwater, FL, United States; 5 The University of Hong Kong Faculty of Education, Hong Kong, Hong Kong SAR, China; 6 Department of Cardiology, University of Washington, Seattle, WA, United States

**Keywords:** cardiology, cardiometabolic health, effect modifier, exposome, one health, risk factors, systems biology

## Abstract

**Figure:**
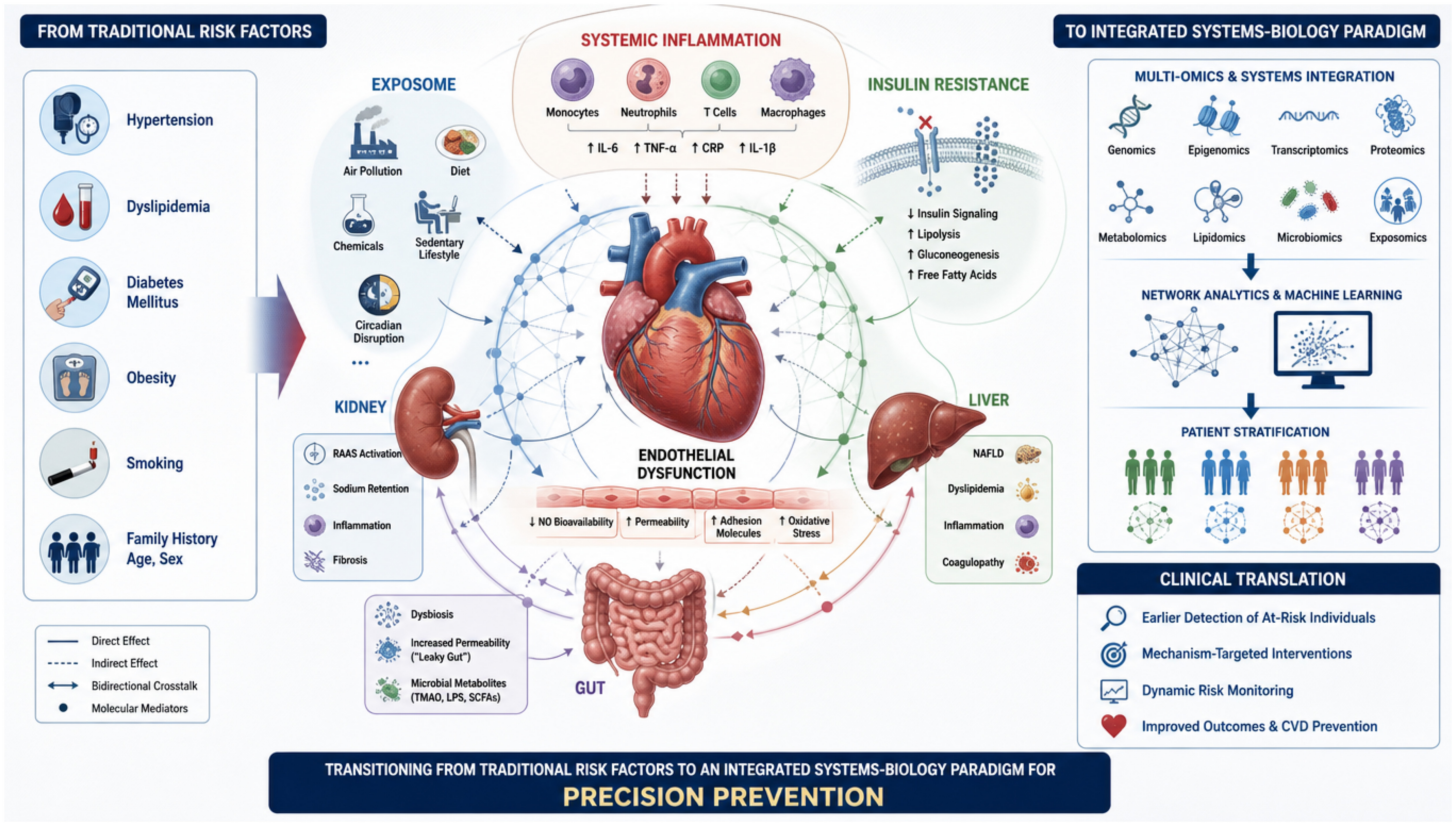
GRAPHICAL ABSTRACT

GRAPHICAL ABSTRACT

Cardiometabolic diseases (CMDs) remain the principal drivers of global morbidity and mortality, despite substantial advances in pharmacotherapy, risk stratification, and preventive cardiology. Conventional epidemiological frameworks—centered on hypertension, dyslipidemia, diabetes, and smoking—have undoubtedly reduced event rates in high-income settings. Yet global trends reveal persistent and, in many regions, accelerating burdens of ischemic heart disease, heart failure, and metabolic dysfunction. The present Research Topic, comprising 36 rigorously conducted research studies, collectively synthesizes that the next phase of cardiometabolic epidemiology must transcend traditional risk enumeration and instead embrace integrative, mechanistic, environmental, and systems-level models of disease.

The conceptual shift evident across these contributions reflects a broader rethinking of cardiovascular vulnerability. Rather than viewing risk factors as isolated exposures, these studies position CMDs as the downstream manifestation of interwoven metabolic, inflammatory, endocrine, environmental, and sociocultural processes. This transition from reductionism to multidimensional epidemiology defines the “new frontier” advanced herein.

## Composite cardiovascular health metrics and mechanistic mediation

Several contributions interrogate composite cardiovascular health indices, particularly Life's Essential 8 (LE8) and Life's Crucial 9 (LC9), in relation to diverse cardiometabolic endpoints. In a large NHANES-based analysis, Li, Ge et al. demonstrated a robust inverse dose–response association between LE8 scores and cardiovascular disease risk, with high scores conferring up to 77% lower odds of total Cardiovascular Disease (CVD). Complementing this, Xu et al. identified a significant inverse association between LE8 and the atherogenic index of plasma, reinforcing the metabolic coherence of the LE8 construct. Extending the framework, Wang, Xu et al. observed that LC9 was inversely associated with congestive heart failure, with estimated glucose disposal rate (eGDR) mediating approximately 66% of this relationship. Tang et al. further linked LC9 to severe abdominal aortic calcification, identifying the systemic inflammatory response index as a partial mediator.

These analyses advance the field beyond associative epidemiology by elucidating intermediary metabolic and inflammatory pathways. Such mediation findings resonate with prior mechanistic frameworks emphasizing insulin resistance and systemic inflammation as central nodes in cardiometabolic progression. Collectively, these studies suggest that composite health metrics exert protective effects not merely through behavioral aggregation but via measurable biological intermediaries.

## Insulin resistance and the centrality of the TyG axis

Among emerging biomarkers, the triglyceride–glucose (TyG) index features prominently across this Topic. Chen, Wen et al. demonstrated that elevated TyG predicted incident CMDs among metabolically healthy obese individuals over 11 years. Chen, Wu et al. (CKM study) further identified a non-linear association between TyG and hyperuricemia in early cardiovascular–kidney–metabolic syndrome. Guo et al. integrated TyG with carotid ultrasound parameters to predict ischemic stroke, achieving an AUC of 0.93 in combined models. Wang, Lu et al. linked TyG to major adverse cardiac and cerebrovascular events following percutaneous coronary intervention, while Li, Fan et al. associated TyG with heart failure with preserved ejection fraction (HFpEF) in coronary heart disease.

These convergent findings elevate TyG from surrogate marker to scalable stratification tool. The consistent observation of dose–response and non-linear patterns suggests a threshold biology, aligning with experimental data linking insulin resistance to endothelial dysfunction, myocardial fibrosis, and prothrombotic states. Importantly, subgroup analyses frequently identified older adults, women, and hypertensive patients as particularly susceptible—underlining the heterogeneity of metabolic risk expression. Furthermore, longitudinal analyses within the Topic further suggested that dynamic metabolic trajectories and evolving adiposity patterns may predict future ASCVD risk more accurately than single-point anthropometric measurements, reinforcing the transition toward time-dependent cardiometabolic risk modeling.

## Re-defining adiposity and ethnic-specific metabolic risk

Traditional reliance on body mass index (BMI) is increasingly challenged by more refined anthropometric and physiologic metrics. Zhang, Shi et al. demonstrated that body roundness index (BRI) outperformed BMI and waist circumference in predicting cardiovascular disease among patients with circadian syndrome. Li, Zhou et al. reported sex-specific associations between cardiometabolic index (CMI) and mortality, with stronger associations observed in women. In a detailed physiologic study, Kho et al. employed dual-energy X-ray absorptiometry, cardiopulmonary exercise testing, and insulin suppression test to show that gold-standard adiposity and fitness measures attenuated ancestral differences in insulin resistance between South Asians and Europeans.

These investigations highlight two critical insights: first, visceral adiposity and metabolic fitness may supersede generalized obesity in risk discrimination; second, ethnic heterogeneity in insulin resistance may reflect measurement limitations rather than intrinsic biological inevitability. Such findings align with prior observations of disproportionate cardiometabolic grisk in South Asian populations at lower BMI thresholds.

## Inflammation, hematologic indices, and prognostic stratification

A notable cluster of studies explores inflammatory and hematologic markers as prognostic tools. Yu et al. identified fibrinogen-to-albumin ratio (FAR) as an independent predictor of long-term mortality among diabetic patients with atherosclerotic cardiovascular disease. Zhu et al. linked blood urea nitrogen-to-albumin ratio (BAR) to cardiovascular and all-cause mortality in diabetes, while Wang, Yang et al. demonstrated that albumin-corrected anion gap predicted cardiovascular and cancer mortality. Song et al. showed that elevated neutrophil-to-lymphocyte ratio independently predicted mortality in patients with coronary heart disease and hypertension, with partial mediation through renal function. Ma et al. observed that red cell distribution width partially mediated the association between smoking and coronary heart disease. Deng et al. identified peripheral thyroid sensitivity (fT3/fT4 ratio) as inversely associated with mortality in metabolic syndrome.

Collectively, these markers capture systemic inflammation, metabolic acidosis, renal impairment, and endocrine dysregulation—illustrating the multi-organ nature of cardiometabolic risk. The frequent identification of non-linear associations underscores the inadequacy of linear risk assumptions in advanced disease states.

## Cardio–renal–hepatic–gastrointestinal crosstalk

The inter-organ axis receives further attention across several studies. Zhang, Jiang et al. used speckle-tracking echocardiography to demonstrate subclinical systolic dysfunction in diabetic nephropathy, linking albuminuria and cholesterol to myocardial impairment. Wang, Ban et al. employed bidirectional Mendelian randomization to identify causal relationships between gastrointestinal diseases—particularly gastroesophageal reflux and celiac disease—and coronary artery disease. Hematillake et al. explored self-management behaviors among Hispanic/Latino patients with metabolic dysfunction-associated steatotic liver disease, emphasizing sociocultural determinants of exercise adherence. Complementing this hepatic–cardiovascular framework, Yan et al. demonstrated an inverse association between serum bilirubin levels and carotid atherosclerosis, further supporting the role of hepatic-metabolic and oxidative pathways in vascular remodeling and atherogenesis. These findings reinforce the cardiovascular–kidney–metabolic (CKM) paradigm, in which metabolic, inflammatory, and structural abnormalities propagate bidirectionally across organ systems.

## Environmental, dietary, and exposomic determinants

A transformative dimension of this Topic is its integration of environmental exposures into cardiometabolic epidemiology. Mao et al. quantified the global burden of cardiovascular disease attributable to air pollution, identifying 2.46 million deaths in 2021 alone. Du et al. demonstrated microclimate-specific thresholds for temperature and pressure fluctuations associated with cerebrovascular events. Li, Liu et al. reported associations between blood metals—particularly lead and cadmium—and cardiovascular disease, employing machine learning models for discrimination. Mao and Kong further examined the global burden attributable to low omega-6 polyunsaturated fatty acid intake. Their projections indicated that while high–Socio-Demographic Index (SDI) regions may experience continued declines in disease burden, countries such as Russia and several nations across North Africa and the Middle East could witness increasing cardiovascular morbidity and mortality attributable to insufficient omega-6 intake by 2030 and 2050. These findings emphasize the enduring importance of dietary fatty acid composition as a modifiable cardiometabolic risk determinant and highlight the necessity for region-specific nutritional and public health interventions. These contributions align with growing evidence linking environmental and dietary exposures to cardiovascular pathology. Collectively, these articles reinforce an emerging paradigm in cardiovascular science: CMDs cannot be fully understood through conventional risk factors alone, but rather through the cumulative biological imprint of environmental, climatic, toxicologic, and nutritional exposures across the life course. The work thus indicates a conceptual shift toward an exposome-informed framework for cardiovascular and metabolic disease research, prevention, and policy.

## Global burden, aging, and predictive modeling

Liang et al. analyzed three decades of Global Burden of Disease data, documenting substantial increases in ischemic heart disease incidence driven primarily by aging and population growth, with projections suggesting further expansion through 2046. Such modeling emphasizes that demographic transitions may offset gains achieved through clinical interventions unless upstream determinants are addressed. The convergence of predictive modeling, mediation analysis, Mendelian randomization, and machine learning across these contributions signals methodological maturation. Epidemiology is increasingly positioned not only to quantify risk but to interrogate causality and identify actionable mediators.

## Toward an integrated cardiometabolic paradigm

Taken together, the 36 studies in this Research Topic redefine cardiometabolic epidemiology across five dimensions: (1) integration of composite health metrics; (2) mechanistic mediation via insulin resistance and inflammation; (3) refined anthropometry and ethnic contextualization; (4) environmental and exposomic incorporation; (5) methodological sophistication through causal inference frameworks.

CMDs are no longer adequately described by cholesterol and blood pressure alone. It reflects an interplay of metabolic dysregulation, inflammatory amplification, endocrine signaling, organ crosstalk, environmental exposures, and social determinants. If epidemiology is to meaningfully reduce global cardiometabolic burden, it must integrate precision biomarkers with planetary health perspectives. The contributions assembled herein represent substantive progress toward that synthesis.

